# Caspr1 Facilitates sAPPα Production by Regulating α-Secretase ADAM9 in Brain Endothelial Cells

**DOI:** 10.3389/fnmol.2020.00023

**Published:** 2020-03-06

**Authors:** Shi-Yu Tang, Dong-Xin Liu, Yuan Li, Kang-Ji Wang, Xia-Fei Wang, Zheng-Kang Su, Wen-Gang Fang, Xiao-Xue Qin, Jia-Yi Wei, Wei-Dong Zhao, Yu-Hua Chen

**Affiliations:** Department of Developmental Cell Biology, Key Laboratory of Cell Biology, Ministry of Public Health, and Key Laboratory of Medical Cell Biology, Ministry of Education, China Medical University, Shenyang, China

**Keywords:** Caspr1, brain endothelial cells, ADAM9, sAPPα, NF-κB

## Abstract

The expression of contactin-associated protein 1 (Caspr1) in brain microvascular endothelial cells (BMECs), one of the major cellular components of the neurovascular unit (NVU), has been revealed recently. However, the physiological role of Caspr1 in BMECs remains unclear. We previously reported the nonamyloidogenic processing of amyloid protein precursor (APP) pathway in the human BMECs (HBMECs). In this study, we found Caspr1 depletion reduced the levels of soluble amyloid protein precursor α (sAPPα) in the supernatant of HBMECs, which could be rescued by expression of full-length Caspr1. Our further results showed that ADAM9, the α-secretase essential for processing of APP to generate sAPPα, was decreased in Caspr1-depleted HBMECs. The reduced sAPPα secretion in Caspr1-depleted HBMECs was recovered by expression of exogenous ADAM9. Then, we identified that Caspr1 specifically regulates the expression of ADAM9, but not ADAM10 and ADAM17, at transcriptional level by nuclear factor-κB (NF-κB) signaling pathway. Caspr1 knockout attenuated the activation of NF-κB and prevented the nuclear translocation of p65 in brain endothelial cells, which was reversed by expression of full-length Caspr1. The reduced sAPPα production and ADAM9 expression upon Caspr1 depletion were effectively recovered by NF-κB agonist. The results of luciferase assays indicated that the NF-κB binding sites are located at −859 bp to −571 bp of ADAM9 promoter. Taken together, our results demonstrated that Caspr1 facilitates sAPPα production by transcriptional regulation of α-secretase ADAM9 in brain endothelial cells.

## Introduction

The blood–brain barrier (BBB) is formed by brain microvascular endothelial cells (BMECs) sheathed by perivascular astrocytes and pericytes, which is critical for maintaining brain homeostasis (Zhao et al., 2015; Sweeney et al., 2016). Over the last two decades, studies demonstrated that BBB does not function independently, but as a key component of the neurovascular unit (NVU), which includes neuron, astrocytes, pericytes, microglia, and the BMECs itself (Rubin and Staddon, 1999; Obermeier et al., 2013; Ben-Zvi et al., 2014).

The amyloid protein precursor (APP) is a transmembrane protein that is primarily processed by two distinct pathways. In the amyloidogenic pathway, APP is sequentially cleaved by β-site APP cleavage enzyme 1 (β-secretase) and γ-secretase complex (De Strooper, 2003), producing amyloid β peptide (Aβ), which is the major constituent of amyloid plaques in brains of patients with Alzheimer disease. The alternative nonamyloidogenic pathway involves the cleavage of APP within the Aβ sequence by α-secretase, generating a soluble N-terminal fragments called soluble amyloid protein precursor α (sAPPα; Thinakaran and Koo, 2008). The APP-derived sAPPα has neurotrophic and neuroprotective properties (Mattson et al., 1993; Ring et al., 2007; Tackenberg and Nitsch, 2019) and also could preclude production of neurotoxic Aβ (Haass and Selkoe, 1993; Tennent et al., 1995). We and others identified that nonamyloidogenic pathway is present in brain endothelial cells (Allinson et al., 2003; Kitazume et al., 2010; Wang et al., 2016); however, the mechanism regulating sAPPα production in brain endothelial cells remains incompletely understood.

Contactin-associated protein 1 (Caspr1) was originally identified as an adhesion molecule in myelinated neurons, forming complex with contactin and neurofascin-155 at paranodes to ensure the propagation of action potentials (Peles et al., 1997; Rios et al., 2000; Bhat et al., 2001). Recently, we identified that Caspr1 is expressed at the luminal side of BMECs and acts as a receptor for bacterial virulence factor to facilitate the penetration of pathogenic *Escherichia coli* through the BBB causing bacterial meningitis (Zhao et al., 2018). In this study, we describe a novel role of Caspr1 in regulating the production of sAPPα in human BMECs (HBMECs). Caspr1 depletion reduced sAPPα release by transcriptional downregulation of α-secretase A disintegrin and metalloprotease 9 (ADAM9) *via* a nuclear factor-κB (NF-κB)–dependent signaling pathway. We thus conclude that Caspr1 facilitates sAPPα production by regulation of ADAM9 in brain endothelial cells.

## Materials and Methods

### Antibodies and Reagents

Anti-Caspr1 (ab34151), anti-ADAM10 (ab1997), anti-ADAM17 (ab39163), anti-p65 antibody (ab106129), anti-p-p65 (S276; ab222494), anti-IKKβ antibody (ab32135), anti-snail antibody (ab229701), and anti-SP1 (ab227383) antibody were purchased from Abcam (Cambridge, UK). Anti-ADAM9 antibody (2099S), anti–phospho-IKKα/β (2697S), anti-IκBα (4812S), and anti–phospho-IκBα (2859S) were from Cell Signaling Technology (Boston, MA, USA). Anti-HIF-1α (NB100-105SS) was from Novus (Littleton, CO, USA). DAPI was from Roche (Basel, Switzerland). Secondary antibodies used for immunofluorescence and Western blot were from Jackson ImmunoResearch Laboratories (West Grove, PA, USA).

### Cell Culture

HBMECs were a generous gift from Dr. K. S. Kim (Johns Hopkins University, Baltimore, MD, USA). HBMECs were cultured in RPMI 1640 medium, with 10% fetal bovine serum (FBS; Hyclone, Logan, UT, USA), 10% Nu-serum (BD Biosciences, Franklin Lake, NJ, USA), 2 mM glutamine, 1 mM sodium pyruvate, 1× nonessential amino acid, and 1 × minimum essential medium (MEM) vitamin. The cells were incubated at 37°C in a 5% CO_2_, 95% air-humidified atmosphere. The 293T cells were cultured in high-glucose Dulbecco modified Eagle medium supplemented with 10% FBS. Cells were incubated at 37°C in 5% CO_2_, 95% air-humidified atmosphere.

### Stable HBMEC Cell Line With Caspr1 Knockout

The single-guide RNA (sgRNA) targeting *Caspr1* gene was designed and synthesized by Obio Technology Corporation (Shanghai, China). The *Caspr1* sgRNA (CTGTATGCACGCTCCCTGGG) was cloned into pLenti-U6-CMV-EGFP vector to obtain the pLenti-U6-Caspr1-gRNA-CMV-EGFP construct. The empty vector was used as a control. HBMECs were cultured and transfected with lentivirus [Multiplicity of infection (MOI) = 20:1] expressing Cas9 (pLenti-CMV-Puro-P2A-3Flag-spCas9; Obio Technology Corporation). After 24-h incubation, puromycin (1 μg/ml) was added to select stable transfected cells. HBMECs stably expressing Cas9 were further transfected with lentivirus containing pLenti-U6-Caspr1-gRNA-CMV-EGFP. The cells were digested with trypsin solution 24 h after transfection and seeded in a 96-well plate using limited dilution method to obtain monoclonal cells. Western blot was used to verify the knockout of Caspr1 in HBMECs. For rescue experiment, the cells were infected with adenovirus encoding the full-length Caspr1 (MOI: 1:20) as indicated.

### RNA Interference

The siRNA targeting to *Caspr1* (5′-GGGUCUUCCUAGAGAAUAUTT3′) was synthesized (Genepharma Corporation, Shanghai, China) and transiently transfected into HBMECs by Lipofectamine 2000 (Invitrogen). The nonsilencing siRNA (5′-UUCUCCGAACGUGUCACGUTT-3′) served as control. Seventy-two hours after transfection, the expression of Caspr1 was analyzed by Western blot to assess the knockdown effects.

### Real-Time Reverse Transcription–Polymerase Chain Reaction

The total RNA isolated with TRIzol reagent (Invitrogen, Carlsbad, CA, USA) was reverse transcribed using M-MLV reverse transcriptase (Promega, Madison, WI, USA). Real-time polymerase chain reaction (PCR) was performed on an ABI 7500 real-time PCR system (Applied Biosystems, New York, NY, USA) with an SYBR premix Ex *Taq* kit (Takara Biotechnology, Osaka, Japan) according to the manufacturer’s instructions. The primers for ADAM9, ADAM10, and ADAM17 are listed in Supplementary Table S1. The amplification conditions were as follows: 95°C for 30 s and 40 cycles of 95°C for 5 s, and 60°C for 34 s. The comparative cycle threshold (Ct) method was used to calculate the relative gene expression level, with GAPDH as the internal control. The products of real-time PCR were analyzed on agarose gel electrophoresis and verified by DNA sequencing.

### Western Blot

The experimental procedure of Western blot was performed as described previously (Zhao et al., 2018). Briefly, The cells were lysed with radioimmune precipitation assay buffer (Beyotime, Nantong, China) containing protease inhibitors. The protein samples were separated by sodium dodecyl sulfate–polyacrylamide gel electrophoresis and then transferred to polyvinylidene difluoride membrane. The blotted membrane was blocked with 5% non-fat milk and incubated with the primary antibody. Then, the blots were incubated with a horseradish peroxidase–conjugated secondary antibody. Immunoreactive bands were visualized by Super Signal West Pico chemiluminescent substrate using a Tanon-5200 imaging system (Tanon, Shanghai, China).

### Immunofluorescence

HBMECs grown on coverslips were washed with phosphate-buffered saline (PBS) and fixed with 4% paraformaldehyde. Fixed cells were permeabilized with 0.2% Triton X-100 and then blocked with 5% bovine serum albumin) in PBS. Then, the cells were stained with antibody against p65 (1:100 dilution) and then incubated with secondary antibody conjugated with Alexa594 (1:200 dilution; Invitrogen). Following DAPI staining, the coverslips were mounted and analyzed under confocal laser scanning microscopy (Zeiss LSM880, Zeiss, Jena, Germany).

### Luciferase Reporter Assay

The truncated sequences of ADAM9 promoter were amplified by PCR (primers listed in Supplementary Table S2) and cloned into pGL3 basic plasmids (Promega) with *Sac*I and *Xho*I restriction enzyme (Takara Biotechnology). The constructed plasmids were transfected into 293T cell with pRL-TK encoding Renilla luciferase vector. After 48 h, cells were lysed, and the luciferase activity was detected using SpectroMax M5 (Molecular Devices, Silicon Valley, CA, USA). When indicated, the siRNA sequence (GGGUCUUCCUAGAGAAUAUTT) targeting human Caspr1 corresponding to the coding region was transfected into 293T cell using Lipofectamine 2000, and after 48 h, the activity of luciferase was determined. The nonsilencing siRNA sequence (GGGUCUUCCUAGAGAAUAUTT) was used as a control.

### Chromatin Immunoprecipitation

Chromatin immunoprecipitation (ChIP) was performed according to the protocol of the EZ-ChIP kit (Millipore, Billerica, MA, USA). Cells (1 × 10^7^) were cross-linked with 1% formaldehyde, and the chromatin was sonicated into fragments. Then, the fragmented chromatin was incubated with the anti-p65 antibody, with immunoglobulin G served as the control. Successively to the decrosslink, the immunoprecipitated DNA was eluted and subjected to real-time PCR analysis. The primer sequences were 5′-ATGTTAAAAAATGGCGTGAT-3′ (forward) and 5′-AGGCAAACAACAAGATTCTG-3′ (reverse).

### Enzyme-Linked Immunosorbent Assay

The culture supernatants of HBMECs were collected, and the level of sAPPα in the supernatant of HBMECs was determined by the enzyme-linked immunosorbent assay (ELISA) kits (IBL) according to the manufacturer’s instructions. Absorbance at 450 nm was read using SpectroMax M5 (Molecular Devices).

### Isolation of Nuclear Protein

The HBMECs were cultured until confluent, and 1 × 10^7^ cells were prepared to isolate nuclear proteins with Cytoplasmic and Nuclear Extraction Kit (Invent Biotechnologies, Eden Prairie, MN, USA) according to the user manual.

### Statistical Analysis

The quantitative variables are expressed as the mean ± SD. All analyses were performed using GraphPad Prism software (GraphPad Software, CA, USA). Statistical significance between two groups was analyzed by unpaired two-tailed Student’s *t*-test. One-way analysis of variance (ANOVA), followed by the Dunnett test, was used to compare multiple groups. *P* < 0.05 was considered significant.

## Results

### Caspr1 Loss of Function Reduces sAPPα Secretion in Brain Endothelial Cells

**Figure 1:**
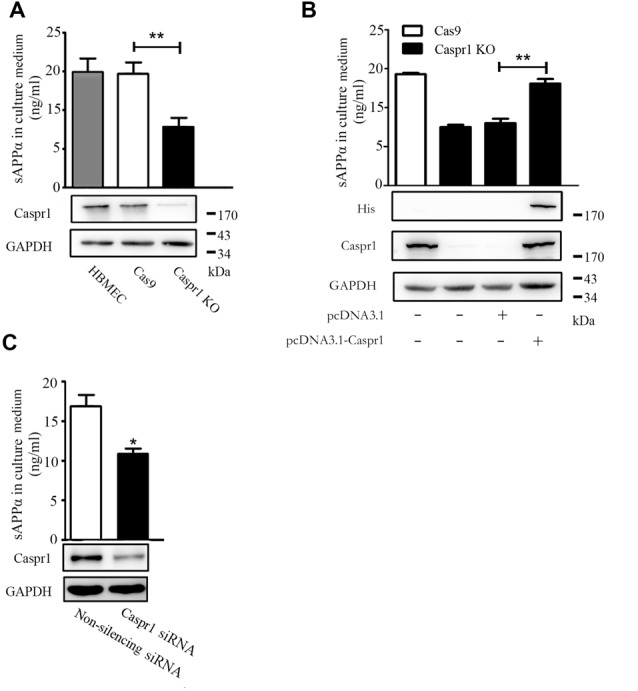
The soluble amyloid protein precursor α (sAPPα) secretion is reduced in brain endothelial cells with loss-of-function of Caspr1. **(A)** The Caspr1 in human BMECs (HBMECs) was knocked out by CRISPR-Cas9 technique verified by Western blot (bottom panel), and then the secreted sAPPα in the medium of Caspr1-depleted HBMECs (Caspr1 KO) was determined by enzyme-linked immunosorbent assay (ELISA; top panel). The empty vector (Cas9) was used as a control. All values are presented as mean ± SD for three independent experiments. **P < 0.01, Student’s *t*-test. **(B)** Stable HBMEC cell line with Caspr1 knockout (Caspr1 KO) was transfected with full-length Caspr1 (with His tag) for rescue experiments, with the empty vector as a control. Caspr1 levels were examined by Western blot (bottom panel). Then, the concentrations of sAPPα in the medium of cells were measured by ELISA. All values are presented as mean ± SD for three independent experiments. **P < 0.01, Student’s *t*-test. **(C)** HBMECs were transiently transfected with Caspr1-specific siRNA, with nonsilencing siRNA as a control. The levels of Caspr1 were examined by Western blot (bottom panel), and the concentrations of sAPPα in the culture supernatant of cells were measured by ELISA. All values are presented as mean ± SD for three independent experiments. *P < 0.05, Student’s *t*-test.

To investigate whether Caspr1 is involved in the processing of APP in brain endothelial cells, we first established stable cell line with knockout of Caspr1 in HBMECs. The sgRNA targeting *Caspr1* gene was designed and cloned into pLenti-U6-CMV-EGFP vector to obtain the pLenti-U6-Caspr1-gRNA-CMV-EGFP construct. Then, the HBMECs stably expressing Cas9 endonuclease were transfected with lentivirus containing pLenti-U6-Caspr1-gRNA-CMV-EGFP to knockout *Caspr1* gene in HBMECs, with empty vector as a control. The stable HBMEC cell lines with Caspr1 knockout were obtained by limited dilution and verified by Western blot analysis. Compared to the control (Cas9), the Caspr1 expression was dramatically reduced in HBMECs with Caspr1 knockout (bottom panel, Figure 1A), and the potential off-target effect of CRISPR was excluded (Supplementary Figure S1A). Then, the sAPPα in the culture medium of Caspr1-knockout HBMECs was measured by ELISA. Interestingly, the results showed that the concentration of sAPPα was significantly decreased in HBMECs with Caspr1 knockout compared to the control (top panel, Figure 1A). The reduction of sAPPα secretion was verified in another stable HBMEC clone with Caspr1 knockout (Supplementary Figure S1B) and by an alternative assay, Western blot analysis (Supplementary Figure S1C). Importantly, when the Caspr1 knockout HBMECs were transfected with the constructs containing full-length Caspr1 cDNA, the reduced sAPPα level was effectively rescued (Figure 1B). To verify these findings, siRNA-mediated knockdown was used to downregulate Caspr1 in HBMECs. The Western blot results showed that the expression of Caspr1 was significantly reduced by the transient transfection of siRNA targeting *Caspr1* compared to the nonsilencing siRNA control (bottom panel, Figure 1C). We further found that the concentration of sAPPα in the culture supernatant was significantly decreased compared to the control (top panel, Figure 1C). These results indicated that Caspr1 is involved in the production of sAPPα in brain endothelial cells.

### Depletion of Caspr1 Downregulates ADAM9 to Suppress sAPPα Production in Brain Endothelial Cells

**Figure 2:**
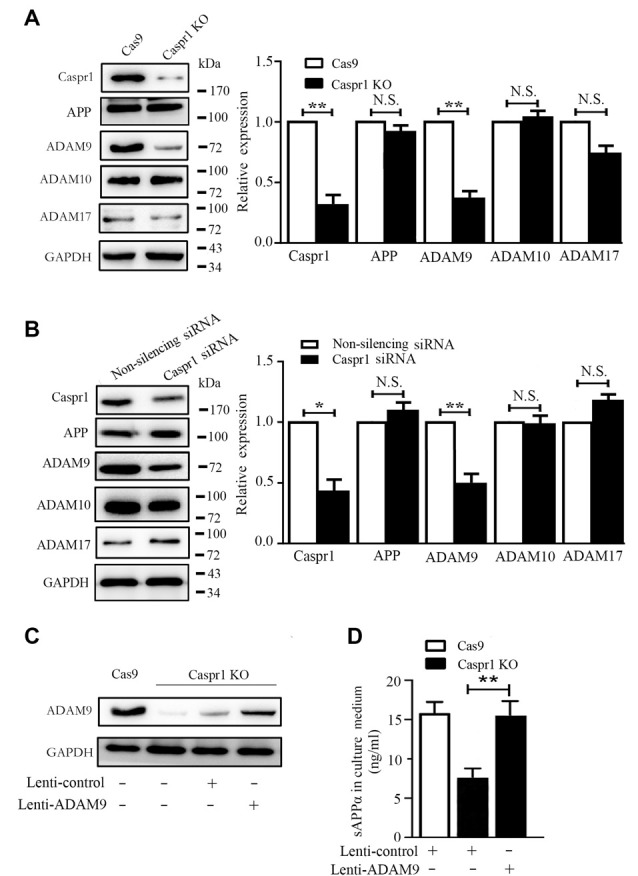
Caspr1 loss of function downregulates ADAM9 to inhibit sAPPα secretion in brain endothelial cells. **(A,B)** The expression of Caspr1, amyloid protein precursor (APP), ADAM9, ADAM10, and ADAM17 was detected in Caspr1-depleted HBMECs (Caspr1 KO) **(A)** and HBMECs transfected with Caspr1 siRNA **(B)** by Western blot, with GAPDH served as loading control. The representative images were from three independent experiments. For quantification, the protein band intensities of the Western blot images were quantified with ImageJ software (National Institutes of Health, Bethesda, MD, USA). The relative protein expression levels were calculated by normalization with the respective GAPDH bands. Data are normalized to controls (Cas9 control or nonsilencing siRNA control), which were defined as 1. Data are presented as mean ± SD for three independent experiments. *P < 0.05; **P < 0.01, Student’s *t*-test. N.S., no statistical significance. **(C,D)** Caspr1-depleted HBMECs were infected with lentivirus encoding the full-length ADAM9 cDNA, with the empty lentivirus used as control. After 36 h, the expression of ADAM9 was determined with Western blot **(C)**, and the concentrations of sAPPα in the medium of the cells were measured by ELISA (D). **P < 0.01, Student’s *t*-test.

It is known that sAPPα is the proteolytic product of APP cleaved by α-secretases. The α-secretase family members mainly include ADAM9, ADAM10 and ADAM17 (Allinson et al., 2003; Lichtenthaler, 2011). To dissect the mechanism of decreased sAPPα in Caspr1 knockout HBMECs, the expression of ADAM9, ADAM10, and ADAM17 was assessed by Western blot. We found the protein level of ADAM9 was significantly reduced in HBMECs upon Caspr1 deletion, whereas the expression of ADAM10 and ADAM17 remained unchanged (Figure 2A). Similarly, the expression of ADAM9, but not ADAM10 and ADAM17, was specifically decreased in HBMECs transfected with Caspr1-specific siRNA compared to the controls (Figure 2B). It is noteworthy that the expression of APP was hardly affected by Caspr1 knockout or Caspr1 knockdown (the second blots from the top, Figures 2A,B), indicating that Caspr1 may regulate the expression of α-secretase ADAM9 without affecting APP, the precursor of sAPPα. Then, the Caspr1 knockout HBMECs were transfected with lentivirus containing the full-length ADAM9 cDNA to restore the expression of ADAM9 (Figure 2C), and the concentrations of sAPPα in the culture supernatant were measured by ELISA. The results showed that the reduced sAPPα secretion upon Caspr1 depletion was effectively rescued by exogenously expressed ADAM9 (Figure 2D), but not by ADAM10 (Supplementary Figure S1D). These data demonstrated that ADAM9 is essential for the Caspr1-regulated sAPPα secretion in brain endothelial cells.

### ADAM9 Is Transcriptionally Downregulated by Caspr1 Depletion

**Figure 3:**
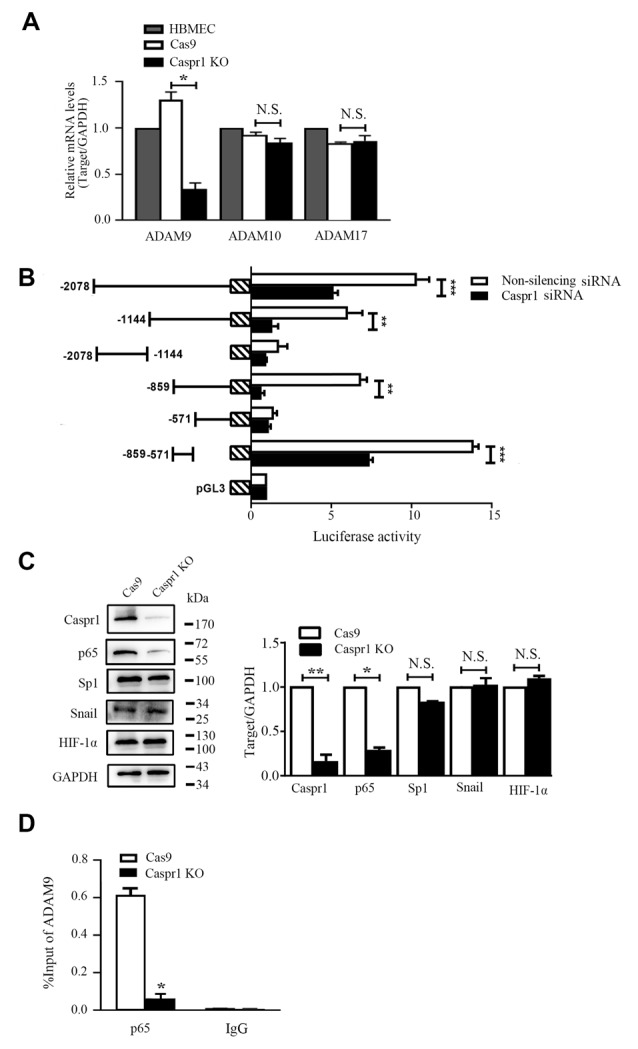
Caspr1 depletion downregulates ADAM9 mRNA levels. **(A)** The total RNA of HBMECs with Caspr1 knockout was extracted and reverse transcribed using Moloney murine leukemia virus (M-MLV) reverse transcriptase. Then, real-time polymerase chain reaction (PCR) was performed to detect the mRNA expression levels of ADAM9, ADAM10, and ADAM17, with GAPDH as an internal control. Data are normalized to HBMECs, which was defined as 1. Values are mean ± SD from three independent experiments. *P < 0.05, Student’s *t*-test. N.S., no statistical significance. **(B)** The truncated sequences of ADAM9 promoter were cloned into pGL3 vector encoding luciferase, respectively. The constructs were then transfected into 293T cells together with pRL-TK plasmids, followed by transfection with Caspr1 siRNA, with nonsilenceing siRNA as control. After 48 h, the cells were harvested and analyzed by luciferase reporter assay. *P < 0.05. **P < 0.01, ***P < 0.001, one-way analysis of variance (ANOVA). **(C)** The expressions of transcription factors including p65, Sp1, snail, and HIF-1α were detected by Western blot in HBMECs with Caspr1 knockout. GAPDH served as loading control. The representative images were from three independent experiments. For quantification, the protein band intensities of the Western blot images were quantified with ImageJ software. Data are normalized to Cas9 controls, which were defined as 1. Data are presented as mean ± SD for three independent experiments. **(D)** Caspr1-depleted HBMECs were subjected to ChIP assay using the NF-κB p65 antibody, whereas the isotype immunoglobulin G served as the control. The immunoprecipitated DNA fragments were amplified by real-time PCR using the primers flanking the promoter regions of ADAM9 genes. The expression levels were quantified, and the statistical analyses were performed. *P < 0.05, Student’s *t*-test.

To further determine the mechanism of decreased ADAM9 in HBMECs with Caspr1 knockout, the mRNA levels of ADAM9 were analyzed by real-time reverse transcription (RT)–PCR. We found the transcripts of ADAM9 were significantly reduced in Caspr1-deleted HBMECs compared to the control, whereas the mRNA expression of ADAM10 and ADAM17 remained unchanged (Figure 3A and Supplementary Figure S1E). These results indicated that Caspr1 specifically regulates ADAM9 expression at transcriptional level. Then, we attempted to dissect the essential region in the promoter of ADAM9 regulated by Caspr1. The truncated DNA sequence of ADAM9 promoter was cloned into pGL vector encoding luciferase. The constructs were then transfected into 293T cells together with pRL-TK plasmids followed by luciferase reporter assay. The results showed that the −859 bp to −571 bp region of ADAM9 promoter contained the necessary elements for adequate expression of ADAM9 (Figure 3B). Furthermore, when the expression of Caspr1 was knocked down by siRNA, the luciferase activity of the critical fragment (−859 bp to −571 bp) of ADAM9 promoter was significantly reduced compared to the nonsilencing siRNA control, which is comparable to the effect observed with the whole −2,078 bp ADAM9 promoter (Figure 3B). Several transcription factors, including p65, Sp1, Snail, and HIF1α, have been reported to be associated with the transcription of ADAM9 (Szalad et al., 2009; Li et al., 2015; Chang et al., 2017; Liu et al., 2017). Then, we analyzed whether the protein levels of these transcription factors were altered in Caspr1 knockout HBMECs. The results showed that the p65 subunit of NF-κB transcription complex was clearly decreased by Caspr1 knockout, whereas Sp1, Snail, and HIF1α were barely affected (Figure 3C). Furthermore, ChIP assay was used to analyze the recruitment of NF-κB to the promoter of ADAM9 gene. We found that the binding of the p65 subunit of NF-κB to the key fragment (−859 bp to −571 bp) of ADAM9 promoter was significantly reduced in Caspr1 knockout HBMECs compared to the control (Figure 3D). These results demonstrated that Caspr1 regulates the mRNA transcription of ADAM9 by the binding of NF-κB with the certain region (−859 bp to −571 bp) of ADAM9 promoter.

### Deactivation of NF-κB Signaling in Caspr1-Depleted Brain Endothelial Cells

**Figure 4:**
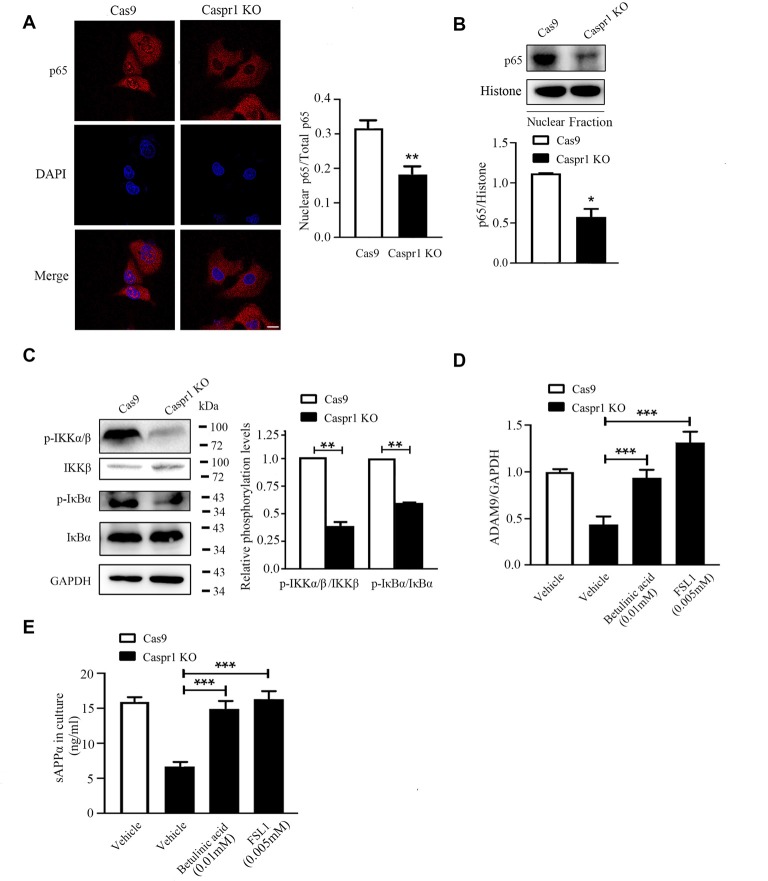
Caspr1 depletion inhibits nuclear factor-κB (NF-κB) signaling pathway. **(A)** The cultured HBMECs with Caspr1 knockout were subjected to immunofluorescence with the primary antibody against the p65 subunit of NF-κB (red). DAPI (blue) was used for counterstaining. The fluorescence intensity of nuclear p65 divided by the p65 in whole cell (total p65) was quantified with ImageJ software. Images are from three independent experiments. Scale, 10 μm. **P < 0.01, Student’s *t*-test. **(B)** The nuclear proteins were extracted from cells, and the levels of the p65 were analyzed by Western blot. The nucleus marker, histone, served as loading control. The representative images were from three independent experiments. For quantification, the protein band intensities of the Western blot images were quantified with ImageJ software. Data are presented as mean ± SD for three independent experiments. *P < 0.05, Student’s *t*-test. **(C)** The activation of NF-κB upstream molecules including IKKβ and IκBα was analyzed by Western blot with antibodies recognizing phosphorylated IKKβ (p-IKKβ) and IκBα (p-IκB). The representative images were from three independent experiments. For quantification of the phosphorylation levels, the protein band intensities of the phosphorylated protein were divided by that of the total protein. Data are normalized to Cas9 controls, which were defined as 1. Data are presented as mean ± SD for three independent experiments. *P < 0.05, Student’s *t*-test. **(D,E)** The HBMECs with Caspr1 knockout were stimulated with NF-κB agonist FSL1 and betalinic acid. **(D)** After 24 h, the total RNA was extracted and reverse transcribed using M-MLV reverse transcriptase. Then, real-time PCR was performed to detect the mRNA expression levels of ADAM9, with GAPDH as an internal control. Data are normalized to HBMECs, which was defined as 1. **(E)** The supernatant of HBMECs was collected; the secreted sAPPα was analyzed by ELISA. Values are mean ± SD from three independent experiment. ***P < 0.001, one-way ANOVA.

It is known that the nuclear translocation of the p65 subunit of NF-κB is necessary for the activation of NF-κB (Napetschnig and Wu, 2013). Our immunofluorescence results revealed that Caspr1 knockout significantly reduced the nuclear localization of p65 (Figure 4A). Then, the nuclear fractions of the cells were extracted to analyze the nuclear p65 protein levels by Western blot, and we found a reduction of nuclear p65 in HBMECs with Caspr1 knockout (Figure 4B). The activation of NF-κB is elicited by the phosphorylation of IκBα caused by activation of IKK (IκB kinase) complex (Hacker and Karin, 2006; Rius et al., 2008). To further dissect the upstream signals regulating NF-κB activity, we assessed the activation status of IKK and IκBα in HBMECs with Caspr1 knockout. The Western blot results showed that the phosphorylation of IKKβ, the major IKK catalytic subunit for NF-κB activation (Hacker and Karin, 2006), was reduced in HBMECs upon Caspr1 knockout (Figure 4C). Consistently, the IκBα phosphorylation was significantly decreased by Caspr1 depletion (Figure 4C). These data indicated that NF-κB signaling is deactivated by Caspr1 depletion in brain endothelial cells. Interestingly, when the HBMECs were treated with NF-κB agonists, betulinic acid and FSL1, the reduced ADAM9 mRNA expression (Figure 4D) and sAPPα secretion (Figure 4E) induced by Caspr1 knockout were effectively restored. These results demonstrated that intracellular NF-κB signaling pathway is required for the Caspr1-regulated sAPPα production.

### Expression of Full-Length Caspr1 Can Rescue the Abnormal Signaling Causing sAPPα Reduction in Brain Endothelial Cells With Caspr1 Knockout

**Figure 5:**
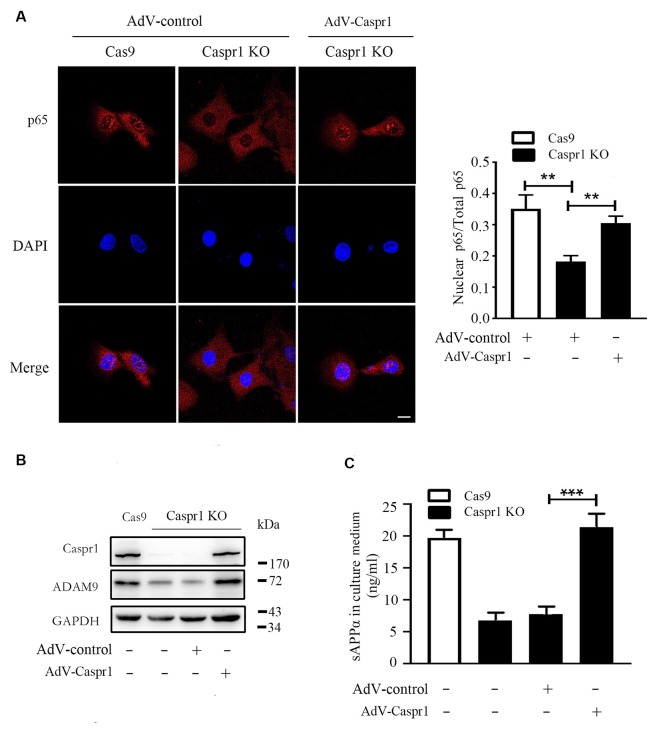
Restoration of Caspr1 expression rescues NF-κB deactivation, ADAM9 downregulation, and sAPPα reduction in Caspr1 knockout cells.** (A)** The HBMECs with Caspr1 knockout (Caspr1 KO) were infected with adenovirus coding the full-length Caspr1 (AdV-Caspr1), with the empty AdV used as a control. The infected Caspr1 KO was subsequently subjected to immunofluorescence with the primary antibody against p65 (red). DAPI (blue) was used for counterstaining. The fluorescence intensity of nuclear p65 was quantified with ImageJ software. Images are from three independent experiments. Scale, 10 μm. **P < 0.01, one-way ANOVA. **(B,C)** The HBMECs with Caspr1 KO were infected with adenovirus coding the full-length Caspr1 (AdV-Caspr1), with the empty AdV used as a control. After 48 h, the ADAM9 expression was examined by Western blot **(B)**. At the same time, the concentration of sAPPα in the medium was measured by ELISA **(C)**. All values are presented as mean ± SD for three independent experiments. ***P < 0.001, one-way ANOVA.

To verify the deactivation of NF-κB signaling is indeed caused by Caspr1 depletion, the Caspr1 knockout HBMECs were infected with adenovirus coding the full-length Caspr1 (AdV-Caspr1), and the nuclear localization of p65 subunit of NF-κB was analyzed by immunostaining. The results showed that the p65 subunit of NF-κB was relocated into the nucleus of Caspr1 knockout HBMECs upon the AdV-Caspr1 infection (Figure 5A). Further results showed that the reduced ADAM9 expression induced by Caspr1 knockout was recovered when the Caspr1-depleted HBMECs were infected with adenovirus containing full-length Caspr1 (Figure 5B). More importantly, the reduced secretion of sAPPα in the Caspr1-deleted HBMECs was restored to normal levels by exogenous expression of full-length Caspr1 (Figure 5C). These results illustrated that, physiologically, Caspr1 promotes ADAM9-mediated sAPPα secretion through the NF-κB signaling pathway in brain endothelial cells.

## Discussion

Our recent studies reported that endothelial Caspr1 acts as a membrane receptor, bound with specific bacterial virulence factors, to facilitate the penetration of pathogenic *E. coli* through the BBB into the brain during bacterial meningitis (Zhao et al., 2018). Upon infection with *E. coli*, Caspr1 receptor can activate intracellular focal adhesion kinase (FAK) signaling to promote rearrangement of actin cytoskeleton for the internalization of bacteria into host endothelial cells (Zhao et al., 2018). However, the physiological function of Caspr1 in brain endothelial cells remained elusive. In this study, our results revealed a novel role of Caspr1 in the regulation of APP processing to produce sAPPα in brain endothelial cells.

The sAPPα is the proteolytic product of APP protein cleaved by α-secretases including ADAM9, ADAM10, and ADAM17 (Allinson et al., 2003; Lichtenthaler, 2011). We found Caspr1 depletion in brain endothelial cells specifically downregulated the expression of ADAM9, without affecting ADAM10 and ADAM17. We further identified that the expression of ADAM9 was regulated by Caspr1 through an NF-κB signaling pathway. These data indicated that Caspr1 has the potential to activate NF-κB signaling to drive ADAM9 expression, leading to cleavage of the APP to produce the soluble sAPPα for secretion. It has been demonstrated that the neuron-derived sAPPα has protective properties against glucose deprivation, glutamate neurotoxicity, and Aβ toxicity (Stein et al., 2004). Thus, the Caspr1-regulated sAPPα derived from brain endothelial cells implies an additional mechanism for protection of neurons, the major component of the multicellular NVU, in the brain.

ADAM9, a type I transmembrane protein, is a catalytically active metalloprotease-disintegrin protein involved in multiple biological processes (Oria et al., 2018; Hsia et al., 2019). Specifically, ADAM9 acts as an α-secretase to cleave APP in nonamyloidogenic pathway producing sAPPα (Koike et al., 1999). The alterations of ADAM9 mRNA levels have been reported in numerous studies, but the transcriptional factors directly regulating ADAM9 expression and the regulatory elements in the ADAM9 promoter remain poorly understood. One study revealed the snail-independent transcription of ADAM9 in lung cancer cell lines, without showing the exact mechanism (Chang et al., 2017). The mRNA and protein levels of ADAM9 were reported to be inhibited by NF-κB inhibitor (Liu et al., 2017), implicating the role of NF-κB in ADAM9 transcription. In this study, we first demonstrated that the p65 subunit of NF-κB can bind with ADAM9 promoter to activate its transcription, which is at the downstream of Caspr1 signaling. The binding region was further characterized as −859 bp to −571 bp of the ADAM9 promoter. Our results thus provided important clues for understanding the molecular mechanisms underlying the transcriptional regulation of ADAM9.

The plasma membrane–anchored Caspr1 is able to recruit FAK molecules to regulate the cortical actin rearrangement upon *E. coli* infection (Zhao et al., 2018). Caspr1 also interacts with the β3 subunit of Na^+^/K^+^-ATPase in endoplasmic reticulum to facilitate its maturation and trafficking to plasma membrane (Zhang et al., 2019). Here, we identified that Caspr1 has transcriptional regulatory activity targeting to the NF-κB–dependent ADAM9 transcription. How does the membrane-associated Caspr1 regulate IKKβ phosphorylation to induce the nuclear translocation of NF-κB? Previous studies showed that FAK is located at the upstream of p65 subunit IKKβ (Funakoshi-Tago et al., 2003; Yurdagul et al., 2016), and it remained to be determined whether FAK could directly bind and phosphorylate IKKβ. We have identified that Caspr1 could recruit FAK in brain endothelial cells (Zhao et al., 2018); thus, we speculated that Caspr1 may regulate the activity of IKKβ *via* FAK signaling, which is an interesting issue that needs further investigation.

In summary, our results revealed a novel role of Caspr1 in sAPPα production of brain endothelial cells. Under physiological condition, Caspr1 is able to activate intracellular NF-κB signaling to drive ADAM9 expression, thus facilitating the cleavage of APP to produce sAPPα for secretion. Further *in vivo* experiments might be necessary to verify our findings obtained from *in vitro* cultured brain endothelial cells.

## Data Availability Statement

All datasets generated for this study are included in the article/Supplementary Material.

## Author Contributions

S-YT conducted most of the experiments. S-YT and D-XL drafted the manuscript. YL, K-JW, X-FW, Z-KS, X-XQ, J-YW, and W-GF performed part of the experiments. Y-HC and W-DZ designed the experiments and wrote the final manuscript.

## Conflict of Interest

The authors declare that the research was conducted in the absence of any commercial or financial relationships that could be construed as a potential conflict of interest.
