# Supplementary material for: Caspr1 Facilitates sAPPα Production by Regulating α-Secretase ADAM9 in Brain Endothelial Cells
Source: Front Mol Neurosci. 2020 Mar 6;13:23. doi: 10.3389/fnmol.2020.00023 (PMC7068801; doi:10.3389/fnmol.2020.00023)
Supplement: Supplementary file 1 [file Data_Sheet_1.pdf]

Supplementary Material

Table S1. Primers for detection of *ADAM* family member expression in HBMECs

Target gene	Amplicon length (bp)	Primer sequence, 5'-3', forward, reverse	Nucleotide position	Genbank accession No.
ADAM9	140	TGTGTCTCCTAGTAGCTTCCT<br>CCATTGTGCACACTTTGGCA	3856 - 3876<br>3995 - 3976	NM_003816
ADAM10	200	TTCCCACAAGGCAGTGTTTT<br>TCCACCAAGACAAGCACTGAA	4621 - 4640<br>4820 - 4800	NM_001110
ADAM17	138	TTTGGTAACGCCACCTGCAC<br>CGGAAAACTGCTCACATCGG	31 - 50<br>168 - 149	NM_003183

Table S2. Primers for plasmid construction of truncated *ADAM9* promoter

Amplified fragments	Primer sequence,<br>5'-3', forward, reverse	Clone vector
ADAM9 (-2078－0）	CCGCTCGAGAATGTCTGAATGCTATTACATA<br>CAAGCTTCTCGGCCGTCCGCAGGTT	pGL3
ADAM9 (-571－0）	CCGCTCGAGAATGTCTGAATGCTATTACATA	pGL3
ADAM9 (-859－0）	CCGCTCGAGAATGTCTGAATGCTATTACATA<br>CCAAGCTTGCCTACAAGGCATCTTTTAAAAAA	pGL3
ADAM9 (-1144－0）	CCGCTCGAGAATGTCTGAATGCTATTACATA	pGL3
ADAM9 (-2078－-1144）	CCTCGAGGAGACAAGTCATTTACTCCATTT<br>CAAGCTTCTCGGCCGTCCGCAGGTT	pGL3
ADAM9 (-859－-571）	CCG CTCGAG ACTGAAGCACAGCAGGTCAAA<br>CC AAGCTT TTAATTGGCTTGGAAAGGG	pGL3

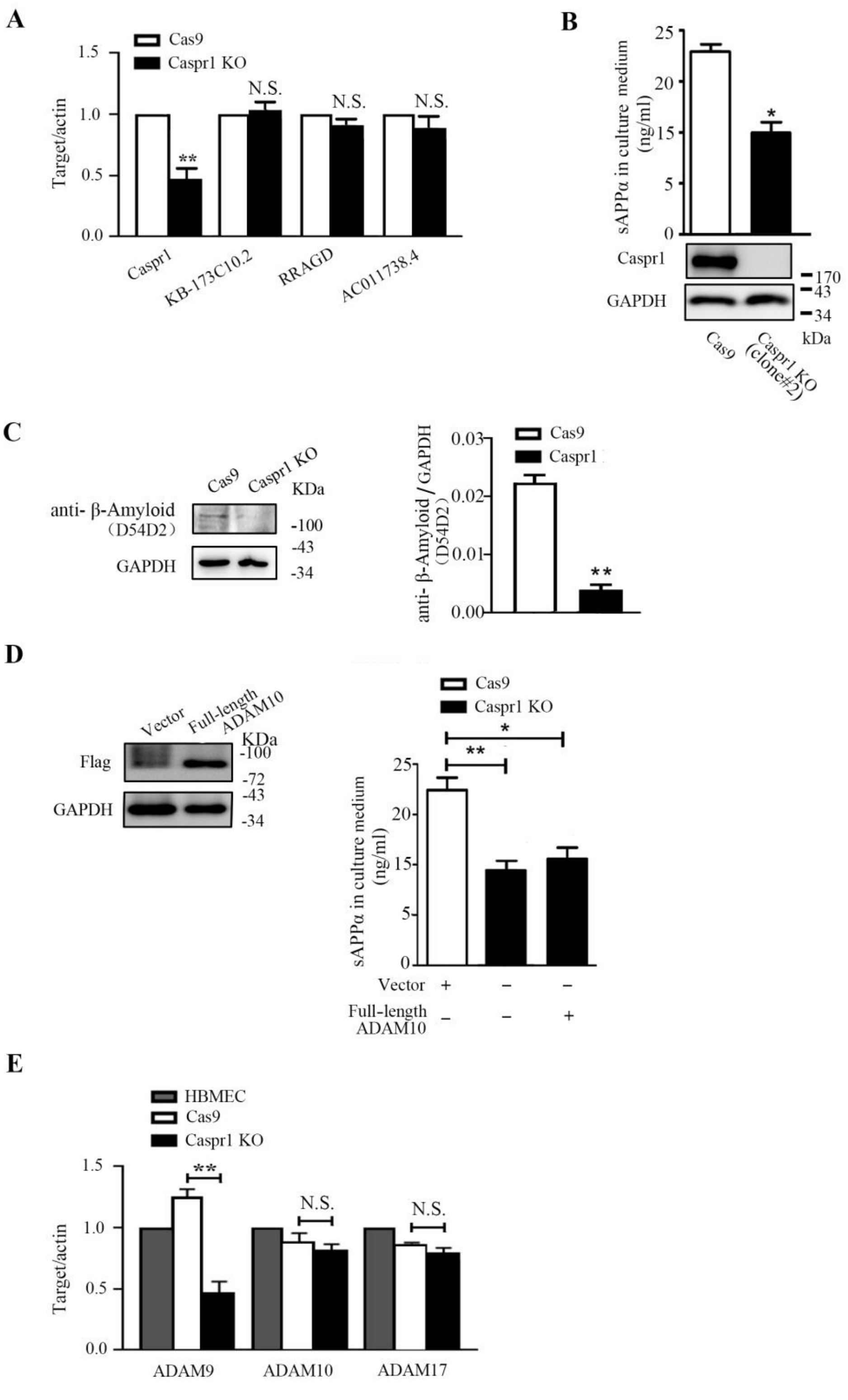

Figure S1. Caspr1 depletion reduced sAPPα production with downregulated ADAM9 in brain endothelial cells

(A) The total RNA of HBMECs with Caspr1 knockout was extracted and reverse transcribed using Moloney murine leukemia virus (M-MLV) reverse transcriptase. Then Real-time PCR was

performed to detect the mRNA expression levels of Caspr1, KB-173C10.2, RRAGD and AC011738.4 genes, with actin as an internal control. Data are normalized to Cas9 control which was defined as 1. Values are mean ± SD from three independent experiments. **, $p<0.01$, Student's t-test. N.S., no statistical significance.

(B) Another single cell clone of HBMECs with Caspr1 knockout by CRISPR-Cas9 technique was obtained and the knockout effect was verified by Western blot (bottom panel). Then the secreted sAPPα in the culture medium of Caspr1-depleted HBMECs (Caspr1 KO) was determined by ELISA assays (top panel). The empty vector (Cas9) was used as a control. All values are presented as mean ± SD for three independent experiments. *, $p<0.05$, Student's t-test.

(C) The expression of sAPPα (~110 kDa) in Caspr1-depleted HBMECs (Caspr1 KO) was detected by anti-β-Amyloid (D54D2) recognizing the C-terminal of sAPPα. The GAPDH was served as loading control. The representative images were from three independent experiments. For quantification, the protein band intensities of the Western blot images were quantified with ImageJ software (National Institutes of Health, Bethesda, MD). The relative protein expression levels were calculated by normalization with the respective GAPDH bands. Data are presented as mean ± SD for three independent experiments. **, $p<0.01$, Student's t-test.

(D) Caspr1-knockout HBMECs were transfected with the construct containing flag-tagged full-length ADAM10 cDNA, with the empty vector as control. After 48 hr, the expression of ADAM10 was determined by Western blot using anti-flag antibody. The concentrations of sAPPα in the medium of the cells were measured by ELISA. *, $p<0.05$, **, $p<0.01$, Student's t-test.

(E) The total RNA of HBMECs with Caspr1 knockout was extracted and reverse transcribed using M-MLV reverse transcriptase. Then Real-time PCR was performed to detect the mRNA levels of ADAM9, ADAM10 and ADAM17, with actin as an internal control. Data are normalized to HBMECs which was defined as 1. Values are mean ± SD from three independent experiments. **, $p<0.01$, Student's t-test. N.S., no statistical significance. The primer sequence of actin was 5'-TGATCTTGATCTTCATTGTG-3' (forward) and 5'-GTTGCTATCCAGGCTGTG-3' (reverse).
